# Variant effect prediction tools assessed using independent, functional assay-based datasets: implications for discovery and diagnostics

**DOI:** 10.1186/s40246-017-0104-8

**Published:** 2017-05-16

**Authors:** Khalid Mahmood, Chol-hee Jung, Gayle Philip, Peter Georgeson, Jessica Chung, Bernard J. Pope, Daniel J. Park

**Affiliations:** Melbourne Bioinformatics, The University of Melbourne, Melbourne, Australia

**Keywords:** Variant effect prediction, Functional datasets, Benchmarking, Mutation assessment, Pathogenicity prediction, Protein function, Functional assays, Genomic screening

## Abstract

**Background:**

Genetic variant effect prediction algorithms are used extensively in clinical genomics and research to determine the likely consequences of amino acid substitutions on protein function. It is vital that we better understand their accuracies and limitations because published performance metrics are confounded by serious problems of circularity and error propagation. Here, we derive three independent, functionally determined human mutation datasets, UniFun, BRCA1-DMS and TP53-TA, and employ them, alongside previously described datasets, to assess the pre-eminent variant effect prediction tools.

**Results:**

Apparent accuracies of variant effect prediction tools were influenced significantly by the benchmarking dataset. Benchmarking with the assay-determined datasets UniFun and BRCA1-DMS yielded areas under the receiver operating characteristic curves in the modest ranges of 0.52 to 0.63 and 0.54 to 0.75, respectively, considerably lower than observed for other, potentially more conflicted datasets.

**Conclusions:**

These results raise concerns about how such algorithms should be employed, particularly in a clinical setting. Contemporary variant effect prediction tools are unlikely to be as accurate at the general prediction of functional impacts on proteins as reported prior. Use of functional assay-based datasets that avoid prior dependencies promises to be valuable for the ongoing development and accurate benchmarking of such tools.

**Electronic supplementary material:**

The online version of this article (doi:10.1186/s40246-017-0104-8) contains supplementary material, which is available to authorized users.

## Background

Screening the entire protein-coding compartment of the human genome yields thousands of protein amino acid substitutions per individual, the majority of which are present at low frequencies (minor allele frequency (MAF) <0.1%) within the population [1]. Genetic screens typically seek to classify variants and genes of relevance to given phenotypes, including disease states. To this end, it is desirable to know whether a given variant is likely to impact protein function, with the inference being that this might influence phenotypes of interest [2–5]. However, appropriate functional assays exist for only a minority of proteins, and in those cases where functional assays do exist, their associated resource requirements are often prohibitive to routine, large-scale application.

Widely used variant effect prediction methods include SIFT [6, 7], PolyPhen (v2) [8, 9], GERP++ [10, 11], Condel [12], CADD [13], fathmm [14], MutationTaster [15], MutationAssessor [16, 17], GESPA [18] and, more recently, REVEL [19]. These use information, variously, about local sequence phylogenetic conservation, amino acid physicochemical properties, functional domains and structural attributes (Table 1). Ensemble or consensus methods such as fathmm, Condel, CADD and REVEL integrate and weight predictions from collections of tools. Recent approaches to algorithm training have applied machine learning techniques. Training and validation (or ‘benchmarking’) of these algorithms has been conducted using datasets that list variants with assigned classifications. Commonly used datasets include HumDiv [20], HumVar [21], Humsavar [22], EPS [23], dbSNP [24] and HGMD [25].

**Table 1:** Characteristics of the protein variant effect prediction tools assessed in this study. The table indicates their scoring ranges and thresholds, training data, summary information about features and, where applicable, machine learning method

Prediction tool	Score range	Deleterious score cutoff	Training data	Features	Machine learning method
GERP++	−12.0 to 6.17	>0.047	None	Infers conserved or constrained elements from 33 mammalian genomes	–
fitCons	0 to 1	>0.4	None	Functional genomics data mainly sourced from chromatin analysis, e.g. ChIP-seq, and evolutionary conservation data	–
SIFT	1 to 0	<0.05	None	Conservation data (MSA of homologous sequences) and transformed into normalised probability matrix	–
PolyPhen	0 to 1	>0.5	HumVar, HumDiv	Conservation data (MSA of homologous sequences), protein functional domain data and protein structural features	Naïve Bayes classifier
CADD	0 to 35+	>15	Simulated, Swissvar, HumVar	Integrates several annotations into a single score, e.g. SIFT, GERP++, PolyPhen, CPG distance, GC content	SVM
Condel	0 to 1	>0.5		Builds a unified classification by integration output from a collection of tools, e.g. SIFT, PolyPhen	Weighted average normalised scores
REVEL	0 to 1	>0.5	HGMD, EPS	HGMD and rare EPS variants used for training	Random forest
fathmm	0 to 1	>0.45	HGMD, Swiss-Prot	Combines evolutionary conservation with disease-specific protein weights for intolerance to mutation	Hidden Markov models

All of the above algorithms have reported potential merit and are widely used in practice. The original publication of REVEL, for example, reported that when this tool was tested against a set of variants from Clinvar, the resulting area under the receiver operating characteristic curve was an impressive 0.96. However, fundamental problems exist with the manner of the training and benchmarking for this and prior tools, centred primarily on the independence and truthfulness of reference sets. Indeed, the authors of REVEL acknowledged that these issues placed potential limitations on their study.

Grimm et al. [20] described the issue of data circularity and its effect on the assessment of prediction algorithms, explaining the importance of the choice and composition of variant datasets used for training and validation. Type 1 circularity results from substantial overlap between training and testing datasets, leading to artificially inflated apparent accuracy in contexts where variants or genes are well represented in training data and to deflated apparent accuracy in settings where they are poorly represented. Type 2 circularity results from all variants in featured genes having been labelled predominantly as either deleterious or benign. Grimm et al. postulated a third type of circularity. In this case, prediction tools contribute to new variant classifications, which, in turn, are used in further benchmarking. Variant classifications within training and benchmarking datasets have been guided substantially by computational predictions, resulting in imperfect ‘truth sets’. Disease risk inflation has been observed in the Clinvar and HumVar databases, whereby considerably fewer individuals in the general population are afflicted with given diseases than would be expected based on pathogenicity classifications within these clinical databases [1]. Ideally, mutation effect prediction training data for supervised machine learning methods should have good coverage of the protein landscape and mutation categorisation that is based on strong evidence from protein functional studies [26]. Miosge et al. [27] reported that of all the amino acid-substituting mutations predicted by PolyPhen to be deleterious to the mouse form of the key tumour suppressor, TP53, 42% had no assay-detectable functional consequence. Similarly, 45% of CADD-predicted deleterious mutations conferred no assay-detectable impact on protein function.

In this study, we conduct benchmarking of eight computational variant impact prediction methods. In addition to assessing their performance using commonly used benchmarking variant datasets, we have derived three independent, functional assay-determined datasets that we have called UniFun (UniProt-derived, functionally characterised, based on UniProt mutagenesis data), BRCA1-DMS (based on deep mutational scanning of BRCA1) and TP53-TA (TP53 mutational scanning via transactivation assay). Our findings have important implications with regard to our confidence in variant classifications derived from computational prediction methods and to how we should train and benchmark such methods in the future.

## Results

**Fig. 1:**
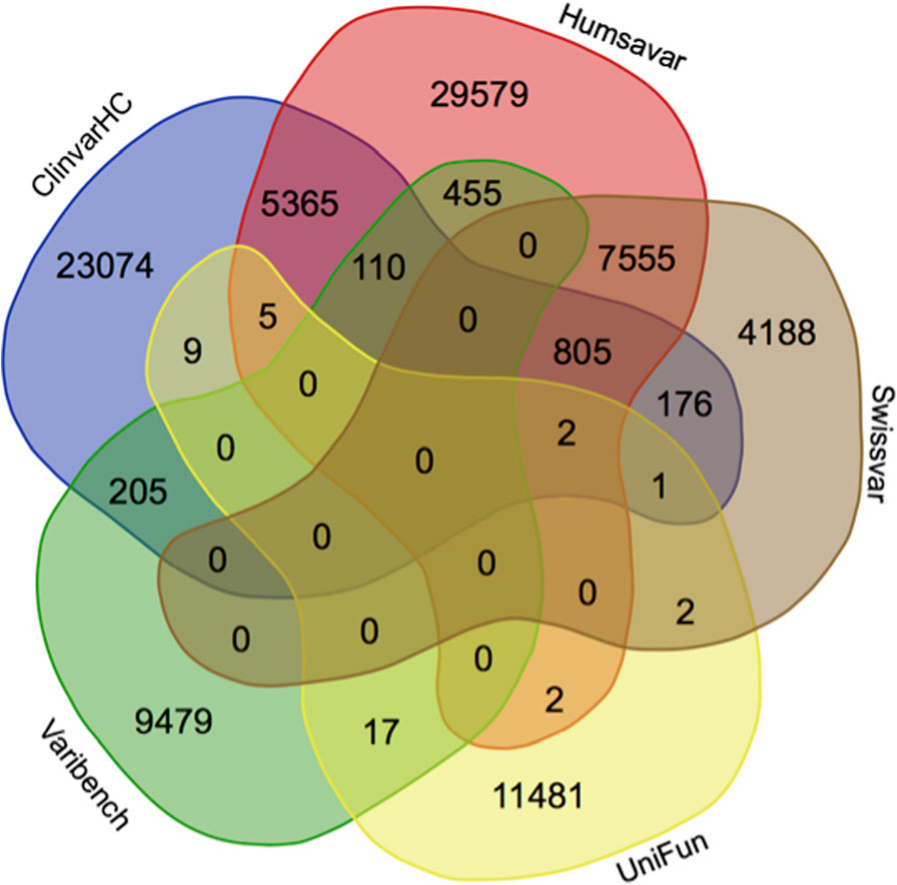
Venn diagram of datasets used in this study showing the overlaps among the deleterious and benign variants observed in these datasets. Humsavar displays a relatively high degree of overlap with the ClinvarHC and Swissvar datasets. The remaining datasets overlap to relatively small extents

In order to limit problems of circularity and systematic error, we derived three human protein mutation datasets that strive for independence from training data and are characterised by direct functional assays: UniFun, BRCA1-DMS and TP53-TA. UniFun represents 11,519 mutations from UniProt for which categorical assignments of protein functional consequence have been made based on direct assays (‘Datasets and methods’). The UniFun variants were sourced from 2209 proteins and exhibit minimal overlap with variants featured in prior disease catalogue datasets (Fig. 1). UniFun is composed of a relatively high percentage of proteins that contribute both deleterious and benign mutations (Additional file 1: Figure S1). BRCA1-DMS (BRCA1 deep mutational scanning) was generated from measured efficiencies of BRCA1 mutants in activities required for efficient homology-directed DNA repair (HDR) and tumour suppression [28]. TP53-TA (TP53 transactivation assay) comprises variants in human TP53 classified by transactivation assay [29] (‘Datasets and methods’) (Table 2).

**Table 2:** Composition of the variant reference datasets used in this study. This table separates mutation catalogues into those derived from clinical databases (disease mutation catalogues) and those derived directly from functional assays (functional mutation catalogues). The table provides summary information for the numbers of proteins and variants of different classifications that have contributed to each dataset. See Additional file 1: Figure S1 and Table S1 for more detailed information

	Total variants	Deleterious	Benign	Total proteins
Disease mutation catalogues				
ClinvarHC	29,752	19,461	10,291	2979
Humsavar	43,878	19,329	24,549	10,231
Swissvar	12,729	4526	8203	5036
Varibench	10,266	4309	5957	4203
Functional mutation catalogues				
TP53-TA	1886	582	1304	1
BRCA1-DMS	1683	408	1275	1
UniFun	11,519	9503	2016	2209

**Fig. 2:**
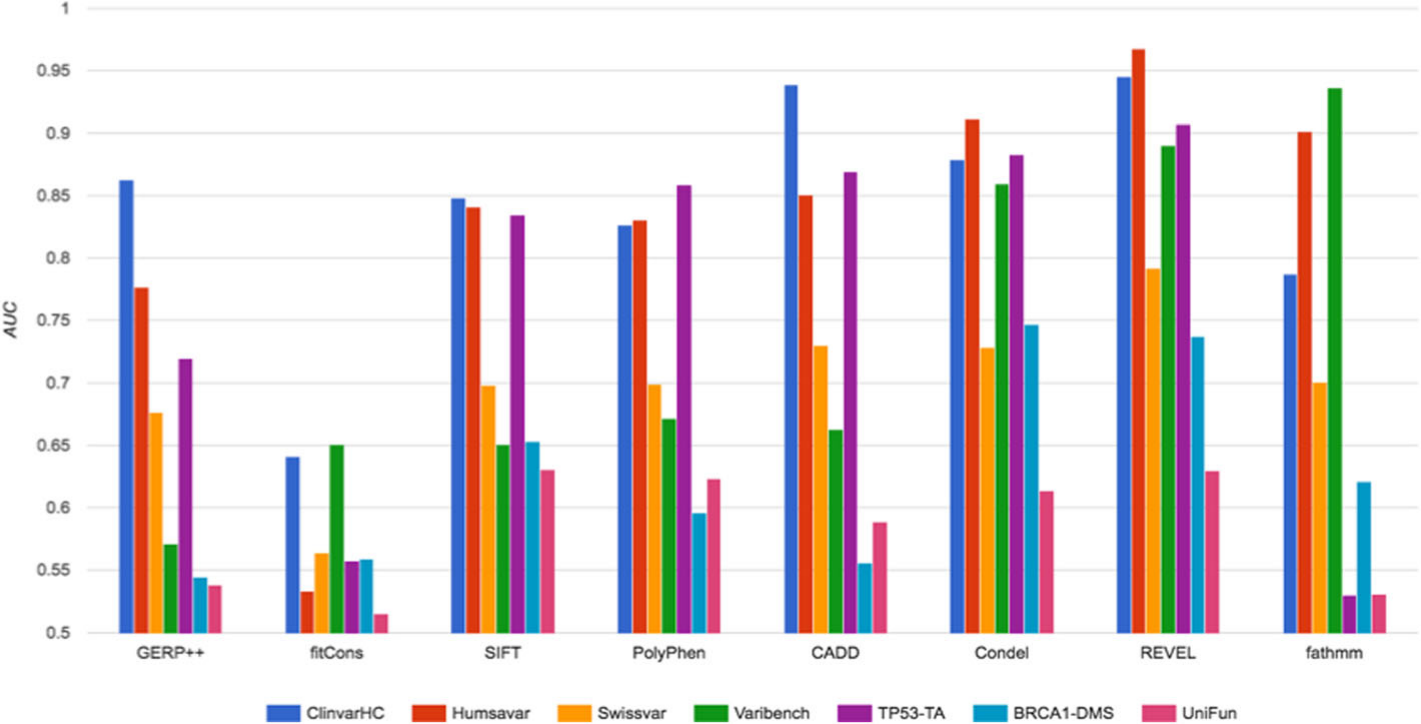
Histogram depicting apparent accuracies of in silico variant effect predictors based on ROC curve AUCs for the benchmarking datasets used in this study

**Fig. 3:**
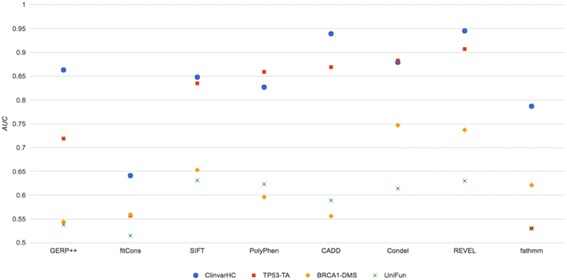
Apparent prediction accuracies of variant effect prediction tools when assessed using ClinvarHC versus functional mutation derived datasets, reported as AUCs derived from ROC curves

Employing the independent, ‘functional’ datasets UniFun, BRCA1-DMS and TP53-TA alongside four prior datasets, we conducted benchmarking of eight prominent variant effect prediction systems (Fig. 2 and Table 3). For all methods, the choice of benchmarking data influenced measured prediction accuracy markedly. When UniFun was employed across tools, the apparent prediction accuracies were consistently among the lowest two measures when compared to measures derived for all the datasets. BRCA1-DMS also tended to yield relatively low apparent prediction accuracies, although the apparent accuracies for Condel, REVEL and fathmm were somewhat elevated when benchmarked using BRCA1-DMS compared with UniFun (see Fig. 3). Compared with UniFun, the BRCA1-DMS and TP53-TA datasets yielded more variable apparent predictive accuracies, with apparent accuracies tending higher for TP53-TA. The apparent prediction accuracies for UniFun, BRCA1-DMS and TP53-TA were in the ranges 0.52 to 0.63, 0.54 to 0.75 and 0.53 to 0.91, respectively. To highlight the strength of influence that the benchmarking dataset choice can have, the apparent accuracy of REVEL, for example, dropped from AUC = 0.945 to AUC = 0.629 when assessment was conducted using UniFun instead of ClinvarHC. For a majority of tools, there was a general grouping of relatively high measured accuracy for the ClinvarHC, Humsavar and TP53-TA datasets. fathmm exhibited its highest apparent accuracy when benchmarked against Varibench (AUC = 0.936), consistent with the observations of [20], and appeared to perform relatively poorly when benchmarked using any of our functional datasets. Remarkably, when benchmarking was conducted using the most extensive and independent of our functional datasets, UniFun, SIFT achieved the highest measured accuracy score of any method tested, at a level comparable with recent machine learning-based methods.

**Table 3:** Measured accuracies of eight in silico predictors as benchmarked against seven different variant reference datasets. Measured accuracies are calculated as the areas under the respective ROC curves (AUCs) and Matthews correlation coefficients (MCCs). See Additional file 1: Figure S4 for the ROC curve graphs

	ClinvarHC		Humsavar		Swissvar		Varibench		TP53-TA		BRCA1-DMS		UniFun	
	AUC	MCC	AUC	MCC	AUC	MCC	AUC	MCC	AUC	MCC	AUC	MCC	AUC	MCC
GERP++	0.863	0.587	0.777	0.469	0.677	0.286	0.571	0.15	0.719	0.283	0.544	0.069	0.538	0.04
fitCons	0.641	0.3	0.533	0.033	0.564	0.008	0.651	0.024	0.557	0	0.559	0	0.515	0.033
SIFT	0.848	0.489	0.841	0.543	0.698	0.289	0.651	0.228	0.835	0.484	0.653	0.199	0.631	0.184
PolyPhen	0.827	0.447	0.831	0.541	0.699	0.301	0.672	0.256	0.859	0.469	0.596	0.088	0.623	0.168
CADD	0.939	0.731	0.851	0.57	0.73	0.331	0.663	0.25	0.869	0.418	0.556	0.032	0.589	0.119
Condel	0.879	0.51	0.911	0.664	0.728	0.333	0.86	0.57	0.883	0.074	0.747	0.172	0.614	0.098
REVEL	0.945	0.68	0.968	0.83	0.792	0.462	0.89	0.59	0.907	0.465	0.737	0.088	0.63	0.148
fathmm	0.787	0.288	0.902	0.538	0.701	0.253	0.936	0.509	0.53	0	0.621	0	0.531	0.02

## Discussion

We have generated three functional datasets that attempt to better represent the truth with regard to variant classifications, guided by direct in vitro functional assays. They are relatively unrelated to prior variant effect prediction tool training datasets. As such, they promise to be useful for tool benchmarking and training, along with similar, expanded datasets in the future. A potential confounder of our functional datasets (although the same applies to other datasets) is that we cannot be certain of our variant classifications—despite being guided by dedicated in vitro tests. Although our functional datasets include genetic variants that have not been observed in human populations to date, observations for established pathogenic mutations support their relevance to the disease setting. Starita et al. [28] showed that for ten known disease-causing missense mutations in BRCA1, all were found to be deleterious by functional assay. Since UniFun represents 2209 proteins, it includes a relatively broad sampling of the human protein landscape and should provide a good basis for general variant effect benchmarking, including for proteins that have not been studied in depth previously. The BRCA1-DMS and TP53-TA datasets focus on single proteins. As single-gene datasets, they do not necessarily offer good representation of the broader protein landscape. They are also likely to be confounded by type 1 circularity because BRCA1 and TP53 are relatively highly represented at the protein level, albeit via different collections of variants, in prior training datasets.

Our observations upon benchmarking a range of in silico variant effect prediction tools against different datasets appeared to broadly reflect the properties of the datasets and how the tools had been calibrated. The high variability of observed prediction accuracies (as measured by the AUC) of the various tools depending on the benchmarking dataset casts serious doubts over the interpretation of outputs from and utility of such tools. That the ‘conservation-only’ tools tended to yield relatively low measured prediction accuracies across datasets is likely due to their comparative naïvety. The low measured prediction accuracies observed when UniFun was used to benchmark machine learning-derived prediction tools are likely to have been influenced by avoidance of circularity problems. This is supported by similar AUC values having been observed by Grimm et al. when they applied their VariBenchSelected and SwissVarSelected datasets, engineered to avoid circularity, to the benchmarking of a variety of variant effect prediction tools.

The general grouping observed for the ClinvarHC, Humsavar and TP53-TA datasets with respect to apparent accuracies of variant effect prediction tools has likely been influenced by substantial protein representation overlaps between these datasets. Approximately 83% of ClinvarHC proteins overlap with Humsavar variant proteins, and TP53 mutations are represented strongly within each of these (Additional file 1: Table S2 and Figure S3). However, BRCA1 variants are even more strongly represented in ClinvarHC and Humsavar than those of TP53, without BRCA1-DMS displaying similar grouping. It is possible that TP53 behaves as a relatively highly representative protein for these datasets with respect to selected predictive features. We observe that a relatively high proportion of TP53 mutants in ClinvarHC and Humsavar are deleterious (Additional file 1: Figure S3). As inferred by Grimm et al., that fathmm’s best apparent performance was observed when benchmarked using Varibench is likely due to type 2 circularity-associated inflation. Varibench contains mutations across 4203 genes, of which only 1.6% have mutations labelled as both benign and deleterious. That SIFT exhibited comparable apparent performance to more recent machine learning-based tools when tested against UniFun may be explained by their training datasets not being conducive to improved general protein effect prediction.

Important to the selection of datasets for training is the issue of whether a prediction tool aims to determine functional consequences generally or only in specific (e.g. particular disease-relevant protein set) contexts. The extent of functional damage conferred by a given variant is an important consideration, which may inform the clinical relevance and preferred classification. Future prediction tools will likely perform best when trained specifically for particular sets of proteins and mutation/variant classes, via multiple partitioned ‘sub-tools’. Regardless, in vitro assay-informed datasets similar to UniFun promise to make important contributions by enabling high-confidence protein functional consequence classifications while allowing training and benchmarking independence.

## Conclusions

Our findings, consistent with those of Grimm et al. [20], indicate that the accuracies of contemporary variant effect prediction tools are likely to be considerably lower than reported in their original method publications. This has profound implications for how we use such tools in clinical diagnostic and disease-gene discovery programs. Indeed, we should treat the predictions generated by such tools with considerable caution. We offer a new paradigm for benchmarking such tools that avoids many of the prior conflicts with the ideals of machine learning. Use of these, and expansion to similar independent, functionally determined mutation datasets as training and benchmarking datasets, will be extremely valuable to the progression of this field. Investigating the properties of incorrectly classified variants and using the findings to better inform algorithm design should result in improved prediction accuracy in the future.

## Datasets and methods

We have employed seven benchmarking datasets (refer to Table 1) to assess the performance of eight amino acid mutation impact prediction methods: GERP++, fitCons, SIFT, PolyPhen, CADD, Condel, REVEL and fathmm. These datasets contain variants classified as *deleterious* (likely significant effect on protein function) or *benign* (unlikely significant effect on protein function). We have used a variety of datasets that can be broadly categorised into two classes: (1) variants sourced from disease variation catalogues and (2) variants sourced from molecular functional analysis experiments. Figure 1 depicts the overlaps between parent datasets used in this study.

### Disease variant catalogues

Databases such as the Swiss-Prot/UniProt-based Humsavar and others, including OMIM [30] and HGMD [25], catalogue disease-associated mutations along with relevant evidence, mainly sourced from the literature. Benign mutations are catalogued via a combination of Swiss-Prot classifications and common alleles (MAF > 1%) from population-based variant databases such as dbSNP and 1000 Genomes.

Clinvar is a database to which contributors submit variants and their classifications along with accompanying evidence. Variously, classifications are based on evidence and assertion criteria such as the Emory Genetics Laboratory Classification Definitions and the InSiGHT Variant Interpretation Committee guidelines [31]. For the present study, we have further filtered Clinvar data to include only high-confidence, expert panel-verified variants with clinical significance scores of 2 (CLNSIG = 2), in the case of benign variants, and 5 (CLNSIG = 5), in the case of deleterious mutations. We term this dataset ClinvarHC (Clinvar high confidence). Mutations classified as likely benign, likely deleterious or of uncertain significance were excluded due to insufficient evidence supporting their influence on protein function and disease.

### ‘Functional’ mutation catalogues

As indicated previously, the disease mutation catalogues in common use for in silico prediction tool training and benchmarking suffer from circularity through a lack of independence on multiple levels [20]. To address this, we have identified that data relating to biochemical assays of protein function, without significant overlap with disease mutation catalogues, should be highly valuable for variant effect prediction tool assessment (and training). These reflect validated effects on protein function while achieving independence. Since highly curated and accessible databases with these properties are not available, we have engineered three such datasets, based on (1) mining functional mutagenesis data from UniProt, (2) the deep mutational scanning (DMS) protocol applied to *BRCA1* and (3) the assessment of TP53 mutants by transactivation assay.

### UniFun dataset

UniFun is derived using protein annotation data from UniProt. In particular, we employed results from human protein mutagenesis experiments in which amino acids had been mutated prior to measuring their effects on protein function. We mined the UniProt data using keywords and the SPARQL querying framework to compose two sets of variants: (1) a ‘functional’ set containing amino acid mutations that disrupt protein function and (2) a ‘non-functional’ set of mutations that have no apparent effect on protein function. More details on how we generated this data are presented in Additional file 1: Figure S2.

### BRCA1-DMS dataset

This relatively new protocol efficiently analyses the impacts of thousands of missense mutations on a protein’s function [32]. Because of the relative recency of this approach, only one publicly available dataset could be sourced [28], derived from measurements of mutated BRCA1 ubiquitin ligase activity and binding to the BARD1 RING domain. Both functions are required for efficient homology-directed DNA repair (HDR) and tumour suppression. The HDR rescue score is used to measure disease risk and is derived from a functional assay to measure the ability of mutant BRCA1 to repair double-stranded DNA breaks. Starita et al. defined an HDR rescue score of 0.53 as the point of inflection between the classifications of deleterious (<0.53) and benign (≥0.53). The authors developed a support vector regression predictor based on both ubiquitin ligase activity and BARD1 RING domain binding to predict the HDR rescue scores for DMS data. In the present study, we employed the conservative approach of categorising variants as ‘deleterious’ if their associated HDR rescue scores were less than 0.33 (above which, no known pathogenic variant score was recorded) and ‘benign’ for HDR rescue scores above 0.77 (below which, the scores of no known benign variants were measured).

### TP53-TA dataset

We sourced unique TP53 amino acid substitutions that had been deposited in the IARC TP53 database [33] (http://p53.iarc.fr) in accordance with the work of Kato et al. [29]. We defined the TP53-TA dataset to exclude variants exhibiting ‘partial’ reduction in transactivation and those variants that are present in the Clinvar and Humsavar databases.

### Data processing

For consistency, Ensembl Variant Effect Predictor (VEP) [34] was employed to convert all variant datasets into variant call format (VCF), using their HGVS amino acid mutation notations as inputs. The resultant VCF files were then annotated using VEP and SnpEff [35]. Condel and fathmm scores were annotated using the VEP custom annotation tools based on precalculated scores available from FannsDB (http://bg.upf.edu/fannsdb). Similarly, GERP++ and fitCons conservation scores were annotated using custom BED files. CADD scores were annotated using CADD v1.2 (http://cadd.gs.washington.edu/download).

### Assessing impact prediction

Prediction performances were evaluated using receiver operating characteristic curves (ROC curves) derived using ratios of true positive rates (TPR or sensitivity) and false positive rates (FPR or 1 − specificity), and the areas under the ROC curves (AUCs) were calculated. AUC values range between 0 and 1, inclusive, where 1 corresponds to a perfect classifier and 0.5 implies a random classification. The Matthews correlation coefficient (MCC) was calculated to measure classifier quality. A score of 1 implies perfect classification and 0 implies random classification.

## Additional file

Additional file 1: Figure S1: Proportion of genes represented in both deleterious and benign variant sets for the respective datasets employed in this study. Figure S2: The UniFun variant dataset is derived from UniProt mutagenesis data (http://www.uniprot.org/help/mutagen). Figure S3: Proportion of deleterious variants for TP53, BRCA1 and the per protein mean in ClinvarHC, Humsavar, Swissvar, Varibench and UniFun variant datasets. Figure S4: ROC curves illustrating the measured performance of eight variant effect prediction methods, GERP++, fitCons, SIFT, PolyPhen, CADD, Condel, REVEL and fathmm, evaluated by seven reference variant datasets: (a) ClinvarHC, (b) Humsavar, (c) Swissvar, (d) Varibench, (e) TP53-TA, (f) BRCA1-DMS and (g) UniFun. Table S1: Protein distribution for deleterious and benign variant classifications across datasets. Table S2: Numbers of variants contributed to the ClinvarHC, Humsavar, Swissvar, Varibench and UniFun datasets by BRCA1 and TP53 and the mean per protein for (a) deleterious variants and (b) benign variants. (DOCX 665 kb)

## Abbreviations

AUC: Area under the curve
BED: Browser extensible data
BRCA1-DMS: BRCA1 deep mutational scanning
ClinvarHC: Clinvar high confidence
FPR: False positive rate
HDR: Homology-directed DNA repair
MAF: Minor allele frequency
MCC: Matthews correlation coefficient
OMIM: Online Mendelian Inheritance in Man
ROC: Receiver operating characteristic
SPARQL: SPARQL protocol and RDF query language
TP53-TA: TP53 transactivation assay
TPR: True positive rate
UniFun: UniProt-derived, functionally characterised
VCF: Variant call format
