# Supplementary material for: Variant effect prediction tools assessed using independent, functional assay-based datasets: implications for discovery and diagnostics
Source: Hum Genomics. 2017 May 16;11:10. doi: 10.1186/s40246-017-0104-8 (PMC5433009; doi:10.1186/s40246-017-0104-8)
Supplement: Additional file 1: — Figure S1: Proportion of genes represented in both deleterious and benign variant sets for the respective datasets employed in this study. Figure S2: The UniFun variant dataset is derived from UniProt mutagenesis data (http://www.uniprot.org/help/mutagen). Figure S3: Proportion of deleterious variants for TP53, BRCA1 and the per protein mean in ClinvarHC, Humsavar, Swissvar, Varibench and UniFun variant datasets. Figure S4: ROC curves illustrating the measured performance of eight variant effect prediction methods, GERP++, fitCons, SIFT, PolyPhen, CADD, Condel, REVEL and fathmm, evaluated by seven reference variant datasets: (a) ClinvarHC, (b) Humsavar, (c) Swissvar, (d) Varibench, (e) TP53-TA, (f) BRCA1-DMS and (g) UniFun. Table S1: Protein distribution for deleterious and benign variant classifications across datasets. Table S2: Numbers of variants contributed to the ClinvarHC, Humsavar, Swissvar, Varibench and UniFun datasets by BRCA1 and TP53 and the mean per protein for (a) deleterious variants and (b) benign variants. (DOCX 665 kb) [file 40246_2017_104_MOESM1_ESM.docx]

Title

Variant effect prediction tools assessed using independent, functional assay-based datasets: implications for discovery and diagnostics

Authors

Khalid Mahmood^1^, Chol-hee Jung^1^, Gayle Philip^1^, Peter Georgeson^1^, Jessica Chung^1^, Bernard J. Pope^1^ and Daniel J. Park^1,*^

Affiliations

1. Melbourne Bioinformatics, The University of Melbourne, Australia

*corresponding author: djp@unimelb.edu.au


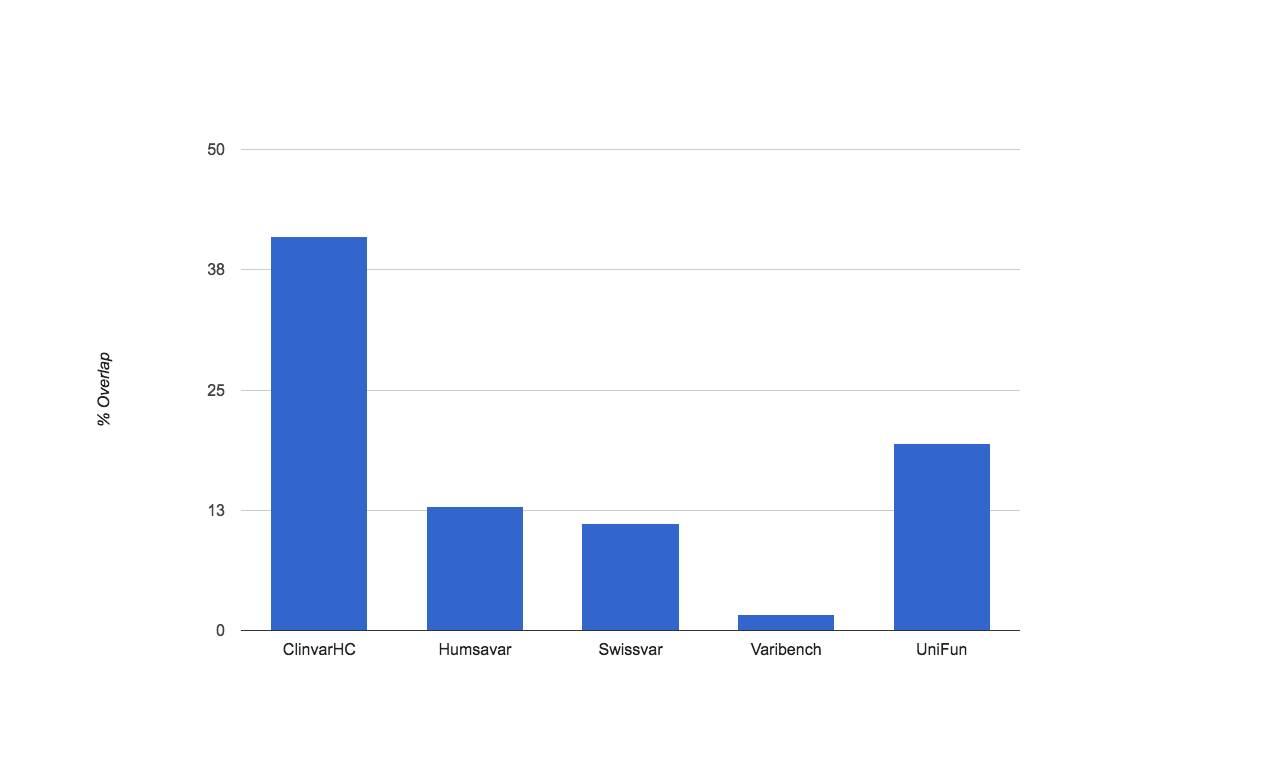


Figure S1: Proportion of genes represented in both deleterious and benign variant sets for the respective datasets employed in this study.

|  | **Genes** | **Deleterious and Benign** | **Deleterious only** | **Benign only** | **Deleterious:Total** |
| --- | --- | --- | --- | --- | --- |
| **ClinvarHC** | 2979 | 1204 | 1492 | 283 | 0.65 |
| **Humsavar** | 10231 | 1320 | 768 | 8143 | 0.36 |
| **Swissvar** | 5036 | 556 | 1248 | 3234 | 0.36 |
| **Varibench** | 4203 | 69 | 211 | 3923 | 0.42 |
| **TP53-TA** | 1 | 1 | 0 | 0 | 0.29 |
| **BRCA1-DMS** | 1 | 1 | 0 | 0 | 0.24 |
| **UniFun** | 2209 | 428 | 1640 | 141 | 0.82 |

Table S1: Protein distribution for deleterious and benign variant classifications across datasets.

| PREFIX up:<http://purl.uniprot.org/core/>  PREFIX taxon:<http://purl.uniprot.org/taxonomy/>  PREFIX faldo:<http://biohackathon.org/resource/faldo#>  SELECT ?protein ?original ?begin ?substitution ?text  WHERE  {  ?protein a up:Protein .  ?protein up:organism taxon:9606 .  ?protein up:annotation ?annotation .  ?annotation a up:Mutagenesis_Annotation .  ?annotation rdfs:comment ?text .  ?annotation up:substitution ?substitution .  ?annotation up:range/faldo:begin/faldo:position ?begin .  ?protein up:sequence ?sequence .  ?sequence rdf:value ?value .  ?annotation up:substitution ?substitution .    BIND( if((?size>0 && ?begin+1<?size),  substr(?value, ?begin, 1),  "NULL"^^xsd:string )  as ?original )    FILTER (CONTAINS(?text, <keywords>)  } |
| --- |

Figure S2: The UniFun variant dataset is derived from UniProt mutagenesis data (<http://www.uniprot.org/help/mutagen>). The UniProt mutagenesis data records the impact of experimental amino acid mutations on the functional properties of the protein. Only point mutation data were used in the formulation of this dataset and longer combinations of point mutations were removed. Each mutation derived from the mutagenesis data was assigned as deleterious or benign based on keywords observed in the experimental description, such as, ‘No effect on function/activity’ refers to a benign mutation while ‘Loss of activity/Abolish function/Loss of function/Abolish of interaction’ refers to a deleterious mutation. The resulting point mutation data were then converted to genomic coordinate based data for further analysis.

a)

| Deleterious | ClinvarHC | Humsavar | Swissvar | Varibench | UniFun |
| --- | --- | --- | --- | --- | --- |
| TP53 | 32 | 70 | 17 | 1799 | 27 |
| BRCA1 | 189 | 23 | 27 | 34 | 9 |
| Mean per protein mean | 7.2 | 9.3 | 2.5 | 15.3 | 4.6 |

b)

| Benign | ClinvarHC | Humsavar | Swissvar | Varibench | UniFun |
| --- | --- | --- | --- | --- | --- |
| TP53 | 9 | 4 | 30 | 0 | 2 |
| BRCA1 | 373 | 14 | 0 | 0 | 7 |
| Mean per protein mean | 6.9 | 2.6 | 2.1 | 1.5 | 3.5 |

Table S2: numbers of variants contributed to the ClinvarHC, Humsavar, Swissvar, Varibench and UniFun datasets by BRCA1 and TP53 and the mean per protein for a) deleterious variants and b) benign variants.


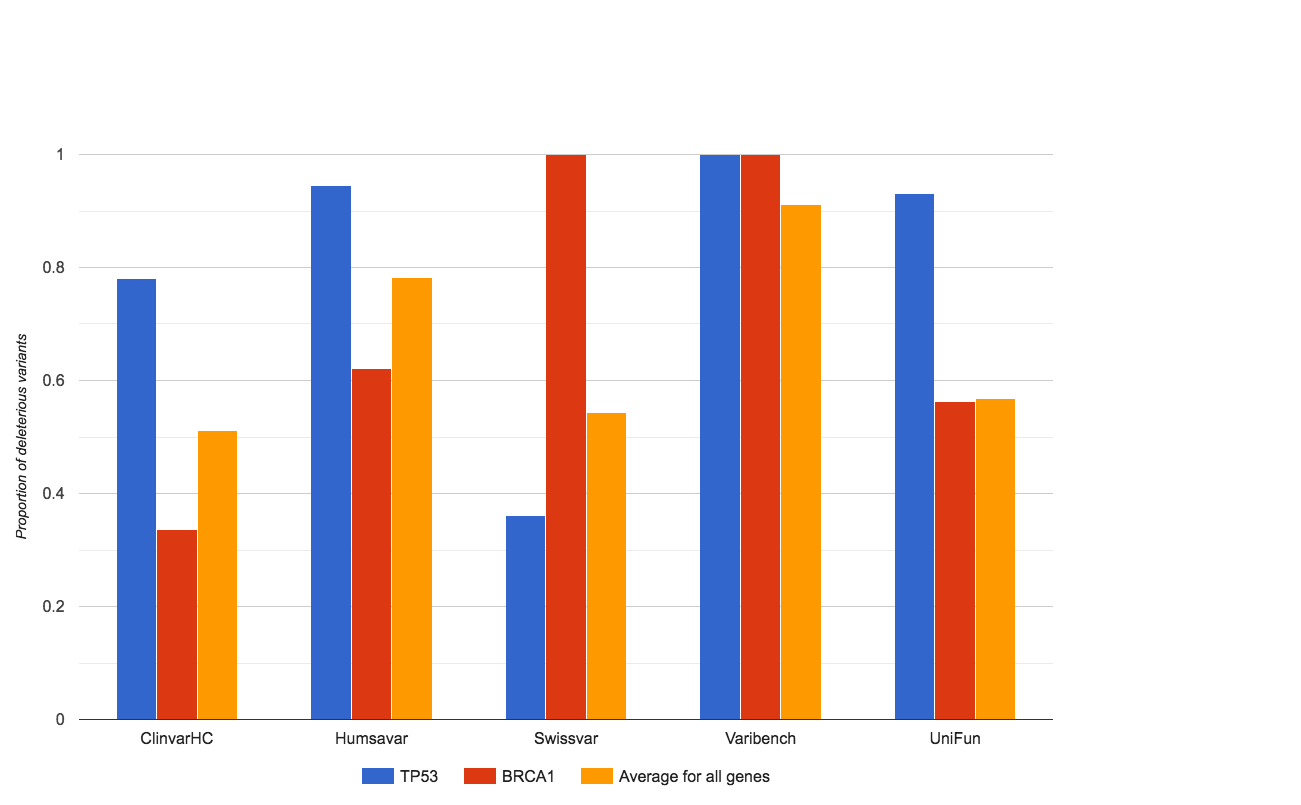


Figure S3: Proportion of deleterious variants for TP53, BRCA1 and the per protein mean in ClinvarHC, Humsavar, Swissvar, Varibench and UniFun variant datasets.

(a)


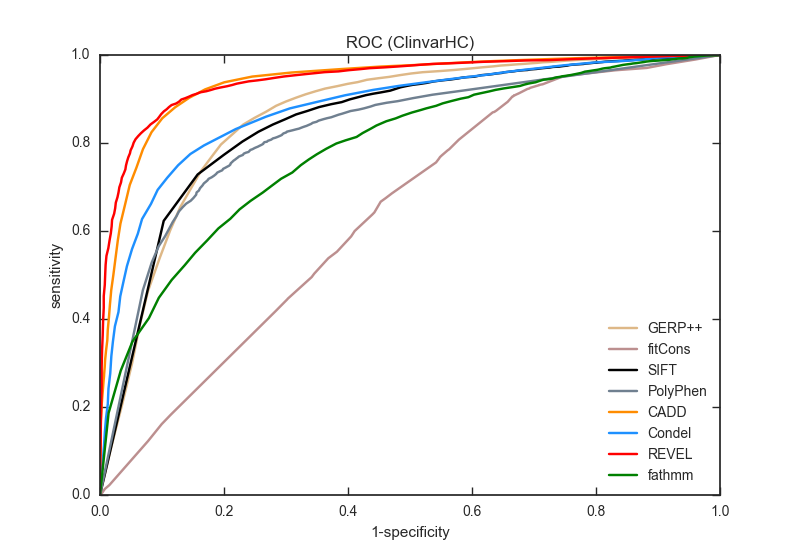


(b)


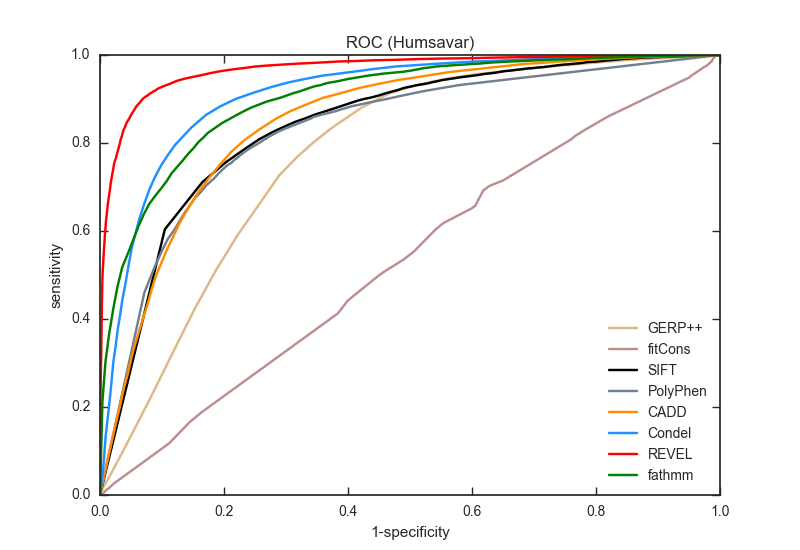


(c)


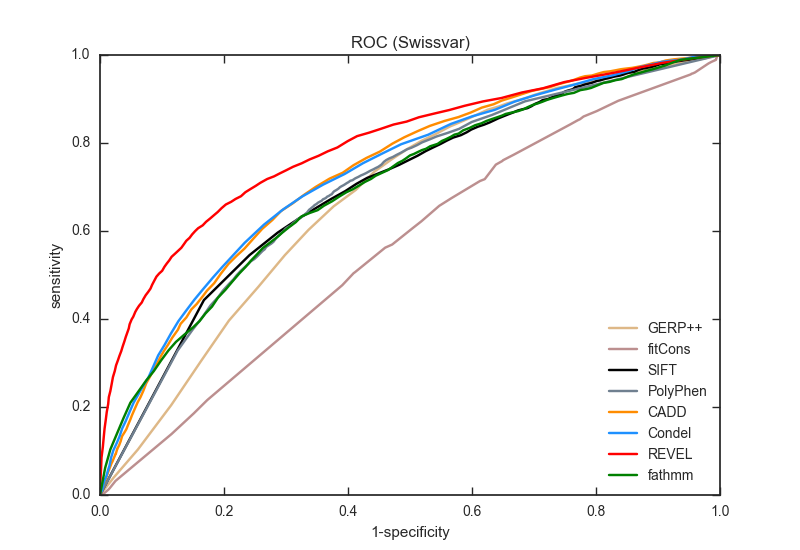


(d)


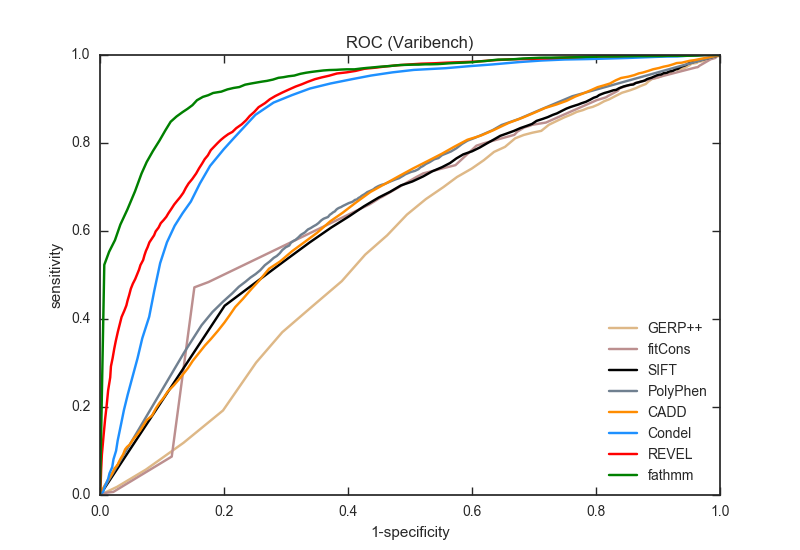


(e)


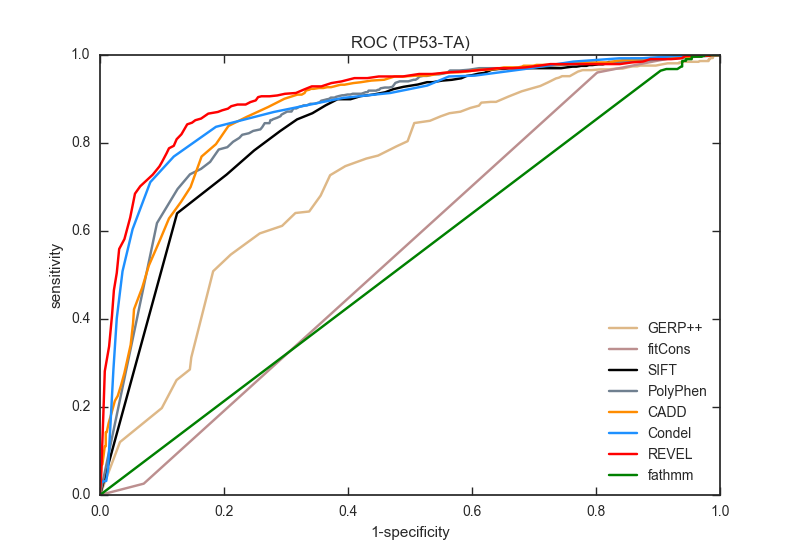


(f)


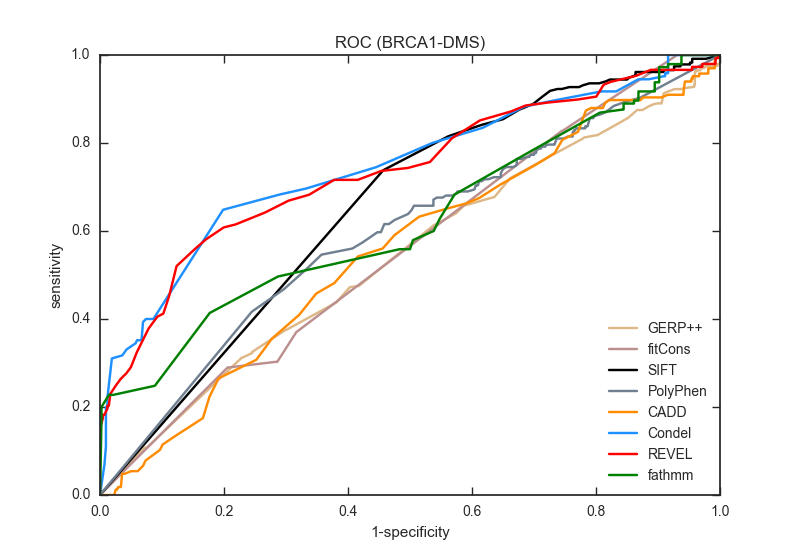


(g)


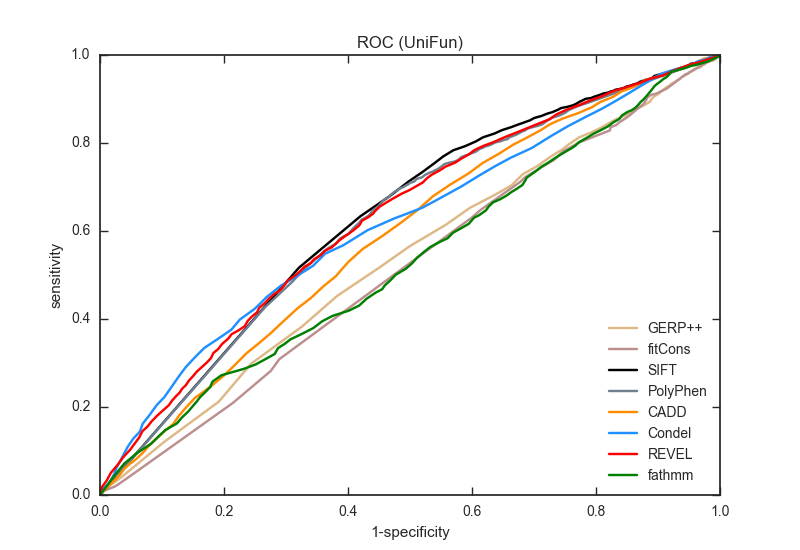


Figure S4: ROC curves illustrating the measured performance of eight variant effect prediction methods, GERP++, fitCons, SIFT, PolyPhen, CADD, Condel, REVEL and fathmm, evaluated by seven reference variant datasets (a) ClinvarHC, (b) Humsavar, (c) Swissvar, (d) Varibench, (e) TP53-TA, (f) BRCA1-DMS and (g) UniFun.
